# A new compact and low phase imbalance microstrip coupler for 5G wireless communication systems

**DOI:** 10.1371/journal.pone.0296272

**Published:** 2023-12-22

**Authors:** Salah I. Yahya, Farid Zubir, Leila Nouri, Zubaida Yusoff, Muhammad Akmal Chaudhary, Maher Assaad, Abbas Rezaei, Binh Nguyen Le

**Affiliations:** 1 Department of Communication and Computer Engineering, Cihan University-Erbil, Erbil, Iraq; 2 Department of Software Engineering, Faculty of Engineering, Koya University, Koya, Iraq; 3 Wireless Communication Centre, Faculty of Electrical Engineering, Universiti Teknologi Malaysia, Johor Bahru, Johor, Malaysia; 4 Institute of Research and Development, Duy Tan University, Da Nang, Vietnam; 5 School of Engineering & Technology, Duy Tan University, Da Nang, Vietnam; 6 Faculty of Engineering, Multimedia University, Persiaran Multimedia, Cyberjaya, Selangor, Malaysia; 7 Department of Electrical and Computer Engineering, Ajman University, Ajman, United Arab Emirates; 8 Department of Electrical Engineering, Kermanshah University of Technology, Kermanshah, Iran; Virginia Military Institute, UNITED STATES

## Abstract

Microstrip couplers play a crucial role in signal processing and transmission in various applications, including RF and wireless communication, radar systems, and satellites. In this work, a novel microstrip 180° coupler is designed, fabricated and measured. The layout configuration of this coupler is completely new and different from the previously reported Rat-race, branch-line and directional couplers. To obtain the proposed coupler, the meandrous coupled lines are used and analyzed mathematically. To improve the performance of our coupler, an optimization method is used. The designed coupler is very compact with an overall size of 0.014λg^2^. The obtained values of S_21_ and S_31_ are -3.45 dB and -3.75 dB, respectively at the operating frequency, while the fractional bandwidth (FBW) is 56.2%. It operates at f_o_ = 1.61 GHz (suitable for 5G applications) and can suppress harmonics up to 2.17f_o_. Another advantage of this coupler is its low phase imbalance, while the phase difference between S_21_ and S_31_ is 180°± 0.023°. Therefore, our device is a balanced coupler with ±0.3 dB magnitude unbalance at its operating frequency. It is important to note that it is very difficult to find a coupler that has all these advantages at the same time. The proposed 180° coupler is fabricated and measured. The comparison shows that the measurement and simulation results are in good agreement. Therefore, the proposed coupler can be easily used in designing high-performance 5G communication systems.

## Introduction

In modern microwave and RF communication systems, microstrip passive devices play a crucial role in signal processing and transmission. These devices include couplers, filters, splitters, and power dividers, among others. Microstrip passive devices are widely used due to their compact size, low cost, ease of fabrication, and excellent performance characteristics. They are used in various applications, such as in satellite communication, radar systems, wireless communication, and medical equipment. In recent years, the demand for high-performance microstrip passive devices has increased significantly due to the growing demand for wireless communication technologies such as 5G, Internet of Things (IoT), and autonomous vehicles [1–9]. Therefore, the development of novel microstrip passive devices with improved performance characteristics is essential to meet the requirements of modern microwave/RF communication systems. Meanwhile, microstrip couplers with small sizes and high efficiency are attractive. Therefore, several types of them have been reported [10–25]. In [10], a microstrip branch-line coupler based on step impedance sections is designed. Two branch-line couplers using microstrip meandrous cells are designed in [11–13]. The proposed coupler in [11] operates at 1.87 GHz for GSM applications. The common advantage of the designed couplers in [11, 13] is their filtering frequency responses. However, the sizes of the reported couplers in [10–13] are large. The presented coupler in [14] is very compact with low phase imbalance. However, it does not has a filtering frequency response. The other microstrip couplers in [15–25] have large implementation areas, while all of them have high phase imbalances. The common weakness of the presented couplers in [10–25] is that their best values of S_21_ and S_31_ are not suitable in their passbands. Two microstrip couplers with arbitrary power divisions and filtering frequency response are designed in [15, 16]. However, they are not able to suppress harmonics correctly. In [17], using T-shape and open stubs a microstrip coupler is designed for 5G applications. In [18], a microstrip three-section branch-line coupler with a wideband is proposed. Interdigital cells are utilized in [19] to obtain a microstrip Rat-race coupler. Another Rat-race coupler using a microstrip spiral resonator is introduced in [20]. To design a 3-dB branch-line coupler in [21], λ/4 open circuited coupled lines are used. The designed coupler in [22] is suitable for 5G applications, which has a simple structure. In this paper, the design of a microstrip 180° coupler is presented to solve the problems of large size, high loss, phase imbalance and harmonics. The configuration of the proposed coupler is completely new and it is not similar to any previous coupler structures. It operates at 1.61 GHz, which is suitable for 5G applications. Our design method is based on a mathematical analysis of a novel resonator. Then, using the proposed resonator two bandpass filters (BPFs) are designed and integrated to obtain the proposed coupler. Additional optimizations are used to improve the frequency response. Finally, to verify the advantages of our coupler a complete comparison with the previous works is done.

## Mathematical analysis of a new resonator

**Fig 1:**
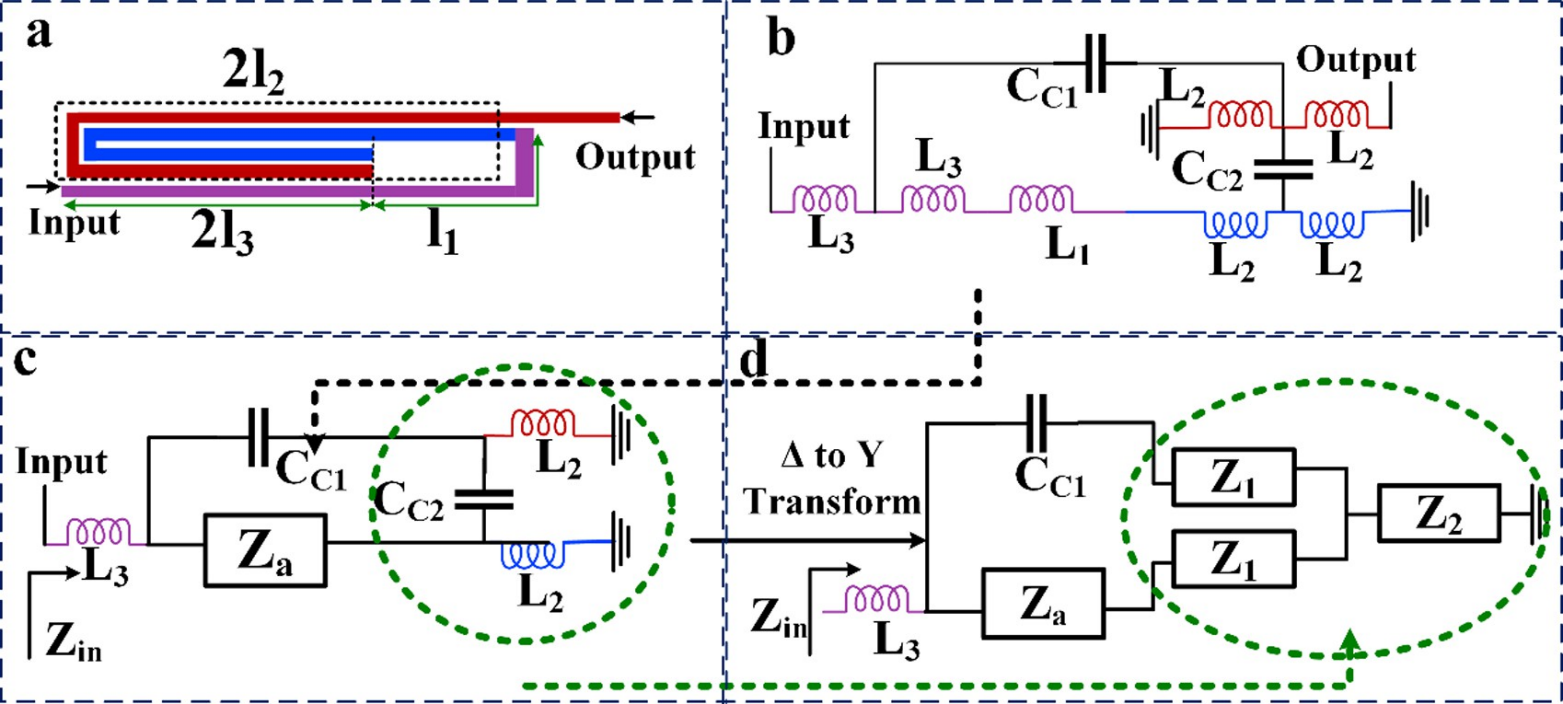
Proposed resonator. (a) layout, (b) equivalent LC circuit, (c) simplified equivalent LC circuit, (d) the Δ to Y transform of the simplified equivalent LC circuit.

Thin coupled lines can create bandpass channels. Because these thin lines have inductive properties and also create some small capacitors. Accordingly, we proposed a novel bandpass resonator as shown in Fig 1. Also, the approximated equivalent *LC* circuit and the simplified *LC* circuit of this resonator are depicted in Fig 1. We ignored the effects of bents in the proposed *LC* circuit. This is because they are significant at frequencies higher than 10 GHz. Also, we used the approximated equivalent of the coupled lines. In the exact model, the number of coupling capacitors will be increased. The coupling capacitors are presented by C_C1_ and C_C2_ in Fig 1. The inductor L_1_ is an equivalent of the line with the physical length l_1_. Meanwhile the equivalents of the physical lengths l_2_ and l_3_ are the inductors *L*_2_ and *L*_3_, respectively. Since there are some microstrip cells with the same physical dimensions, we used similar names for them. For clarity in Fig 1, the colors of the *LC* equivalent circuit are chosen to be the same as its corresponding physical structure. After that, the Δ to Y transform is used to simplify the presented LC circuit. The input impedance is calculated from the input port when the output port is open.

By comparing the equivalent circuits shown in Fig 1(B) and 1(C), it can be seen that the impedance Z_a_ is the sum of the impedances L_1_, L_2_ and L_3_. Therefore, in Fig 1, after simplification of the *LC* circuit the values of the impedance *Z*_*a*_ is:

$$Z_{a}=j\omega \;(L_{1}+L_{2}+L_{3})$$ (1)

Where ω is an angular frequency. As can be seen, *Z*_*a*_ is the impedance of an inductor.

By comparing Fig 1(C) and 1(D), we can see that, the impedances Z_1_ and Z_2_ are the results of Δ to Y transformation, which will be obtained based on the values and locations of C_C2_ and L_2_. Therefore, the values of the impedances *Z*_*1*_ and *Z*_*2*_ can be calculated as follows:

$$Z_{1}=\frac{\frac{1}{j\omega C_{C2}}\times j\omega L_{2}}{\frac{1}{j\omega C_{C2}}+2j\omega L_{2}}\;\Rightarrow \;Z_{1}=\frac{\omega L_{2}}{1-2\omega^{2}C_{C2}L_{2}}j\;\;\;\;(\Omega )$$ (2a)

$$Z_{2}=\frac{j\omega L_{2}\times j\omega L_{2}}{\frac{1}{j\omega C_{C2}}+2j\omega L_{2}}\;\Rightarrow \;Z_{2}=-\frac{\omega^{3}L_{2}^{2}C_{C2}}{1-2\omega^{2}L_{2}C_{C2}}j\;\;\;\;(\Omega )$$ (2b)

By considering the range of the coupling capacitor C_C2_ (C_C2_ has a very small value), the predetermined value of the operating frequency and the range of the inductor L_2_ it can be seen that the impedance Z_1_ belongs to an inductor. Also, as presented in Eq (2B), since *Z*_*2*_ is a negative impedance it can be the impedance of a capacitor. As it can be seen in Fig 1(D), The input impedance of the proposed resonator (*Z*_*in*_) can be obtained by:

$$\begin{array}{l} Z_{in}=j\omega L_{3}+Z_{2}+\frac{\left(Z_{1}+\frac{1}{j\omega C_{C1}})(Z_{1}+Z_{a}\right)}{2Z_{1}+\frac{1}{j\omega C_{C1}}+Z_{a}}\Rightarrow \\ Z_{in}=j\omega L_{3}-\frac{\omega^{3}L_{2}^{2}C_{C2}}{1-2\omega^{2}L_{2}C_{C2}}j+\frac{\left(\frac{\omega L_{2}}{1-2\omega^{2}C_{C2}L_{2}}j+\frac{1}{j\omega C_{C1}})(\frac{\omega L_{2}}{1-2\omega^{2}C_{C2}L_{2}}j+j\omega (L_{1}+L_{2}+L_{3})\right)}{\frac{2\omega L_{2}}{1-2\omega^{2}C_{C2}L_{2}}j+\frac{1}{j\omega C_{C1}}+j\omega (L_{1}+L_{2}+L_{3})}\\ \Rightarrow \;Z_{in}=j\omega L_{3}-j\omega [\frac{\omega^{2}L_{2}^{2}C_{C2}}{1-2\omega^{2}L_{2}C_{C2}}+\frac{\left(\frac{1}{\omega C_{C1}}-\frac{\omega L_{2}}{1-2\omega^{2}C_{C2}L_{2}})(\frac{L_{2}}{1-2\omega^{2}C_{C2}L_{2}}+L_{1}+L_{2}+L_{3}\right)}{\frac{2\omega L_{2}}{1-2\omega^{2}C_{C2}L_{2}}-\frac{1}{\omega C_{C1}}+\omega (L_{1}+L_{2}+L_{3})}] \end{array}$$ (3)

According to the predetermined value of the resonance frequency and also the range of lumped elements, some terms are very small compared to the others. Therefore, the small terms can be removed with a good approximation. Our target frequency is in the GHz range and the inductors are in nH. The simulation results show that the coupling capacitors usually have small values in pF (or fF). Accordingly, to find the resonator behavior we can use the following approximation:

$$1\rangle \rangle 2\omega^{2}C_{C2}L_{2}\;\to \;1-2\omega^{2}C_{C2}L_{2}\;\approx \;1$$ (4)

Based on Eq (4) and the above explanations about the range of lumped elements, Eq (3) can be simplified as follows:

$$\begin{array}{l} Z_{in}\;\approx \;j\omega [L_{3}-\omega^{2}L_{2}^{2}C_{C2}-\frac{\left(\frac{1}{\omega C_{C1}}-\omega L_{2})(2L_{2}+L_{1}+L_{3}\right)}{2\omega L_{2}-\frac{1}{\omega C_{C1}}+\omega (L_{1}+L_{2}+L_{3})}]\Rightarrow \\ Z_{in}\;\approx \;j\omega [\frac{\left(L_{3}-\omega^{2}L_{2}^{2}C_{C2})(\omega^{2}C_{C1}(L_{1}+3L_{2}+L_{3})-1)-(1-\omega^{2}C_{C1}L_{2})(2L_{2}+L_{1}+L_{3}\right)}{\omega^{2}C_{C1}(L_{1}+3L_{2}+L_{3})-1}] \end{array}$$ (5)

To find the operating frequency, we can set *Z*_*in*_ = 0. Therefore, based on the approximated value of Z_in_ in Eq (5) we can obtain the resonance condition as follows:

$$Z_{in}=0\Rightarrow (L_{3}-\omega_{1}^{2}L_{2}^{2}C_{C2})(\omega_{1}^{2}C_{C1}(L_{1}+3L_{2}+L_{3})-1)-\;\;(1-\omega_{1}^{2}C_{C1}L_{2})(2L_{2}+L_{1}+L_{3})=0$$ (6)

By setting the numerator of Eq (5) equal to zero, Eq (6) can be obtained where ω_1_ is the operating frequency of the proposed resonator. Based on the range of inductors, capacitors and angular frequency, the following approximations can be applied in Eq (6):

$$\left\{\begin{array}{l} L_{3}-\omega_{1}^{2}L_{2}^{2}C_{C2}\approx L_{3}\\ \omega_{1}^{2}C_{C1}(L_{1}+3L_{2}+L_{3})-1\approx -1\\ 1-\omega_{1}^{2}C_{C1}L_{2}\approx 1 \end{array}\right.\Rightarrow -L_{3}-(2L_{2}+L_{1}+L_{3})=0\Rightarrow -2L_{3}=2L_{2}+L_{1}$$ (7)

The above approximations are acceptable for the small coupling capacitor (C_C2_ is in fF or pF ranges), the inductor in nH, where *ω*_*1*_ can be a resonance frequency or a harmonic in GHz. Since Eq (7) is not reasonable, we conclude that the resonance frequency can occur when *Z*_*in*_ = ∞. This is an advantage. Because if we have two resonance frequencies, one of them is a harmonic (*ω*_*1*_) that must be removed. But our proposed structure removes this harmonic by itself. For *Z*_*in*_ = ∞, we can write that:

$$Z_{in}=\infty \Rightarrow \omega_{2}^{2}C_{C1}(L_{1}+3L_{2}+L_{3})-1=0\Rightarrow \omega_{2}=\sqrt{\frac{1}{C_{C1}(L_{1}+3L_{2}+L_{3})}}$$ (8)

Where *ω*_*2*_ is the operating frequency of the proposed resonator. Based on Eq (8), the values of inductors and capacitors can be adjusted for a predetermined angular frequency. By adjusting these values, the dimensions of the transmission lines and the distance between the coupled lines can be found. Therefore, it is possible to simultaneously reduce the dimensions and adjust the operating frequency using Eq (8). Using the presented mathematical analysis and calculated angular frequencies, the behavior of the basic resonator is specified and as a result the optimization of the final layout can be done more easily. Based on Eq (8), for a predetermined target resonance frequency, we have a high degree of freedom to choose inductors and coupling capacitors. After choosing them, we can calculate the physical dimensions using the Richard transformations. However, the exact dimensions will be determined after applying the optimization method and changing the important dimensions extracted by the presented mathematical analysis and equations. Using Eq (8), the resonator behavior can be determined. Then we can optimize the dimensions accordingly. For example, the resonance frequency can be determined beforehand. Then, the values of the inductors can be changed in such a way that the least possible space is occupied. A method is reducing the distance between coupled lines to reduce the insertion loss. However, we should be careful not to increase the harmonics by reducing the gap between coupled lines. Then, for two predetermined values of the resonance frequency and the coupling capacitor, the inductors can be determined using the optimization method. According to Eq (8), by increasing an inductor, the operating frequency moves to the left. Therefore, increasing the physical lengths presented in Fig 1(A) (l_1_, 2l_2_, and 2l_3_), shifts the resonance frequency to the left. Using the analyzed resonator and additional optimizations, two bandpass filters (BPFs) i.e. BPF1 and BPF2 are designed.

### Design and analysis of a novel coupler

**Fig 2:**
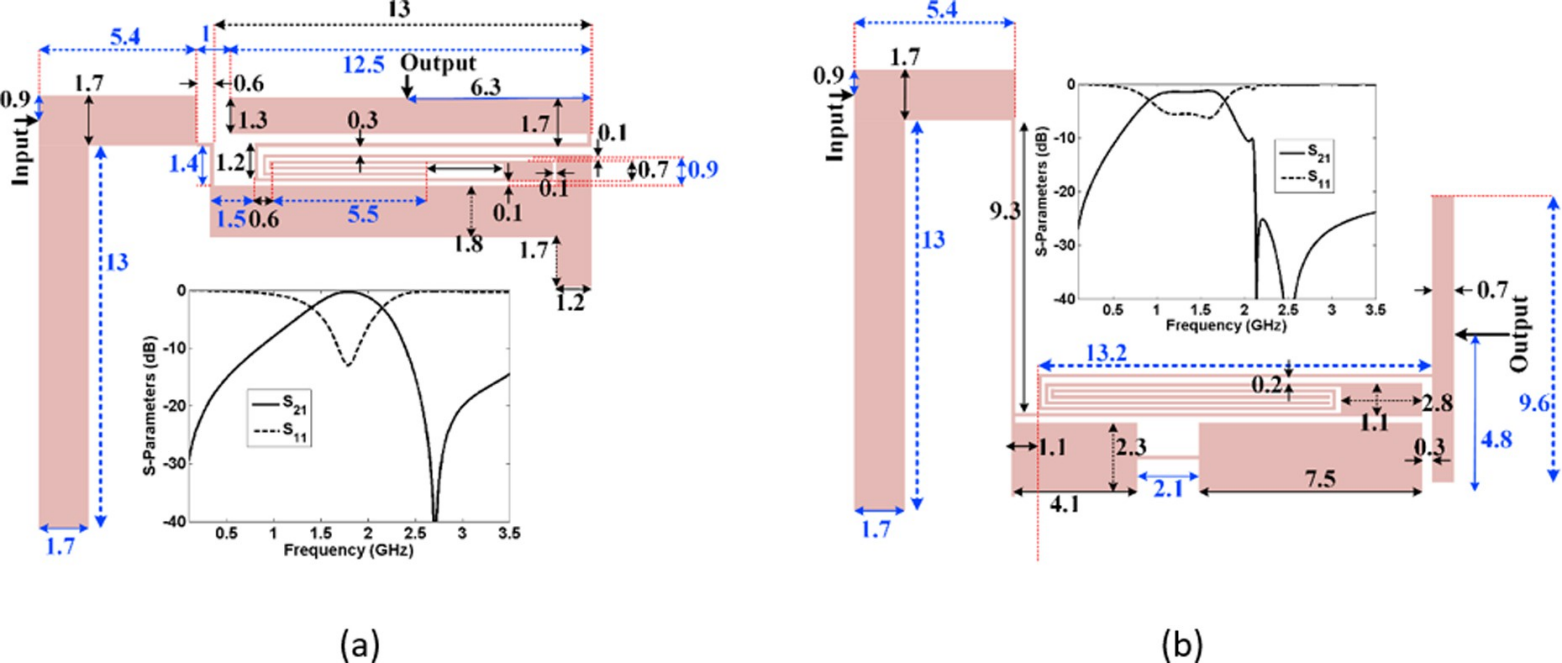
The proposed BPFs and their frequency responses. (a) BPF1, (b) BPF2.

**Fig 3:**
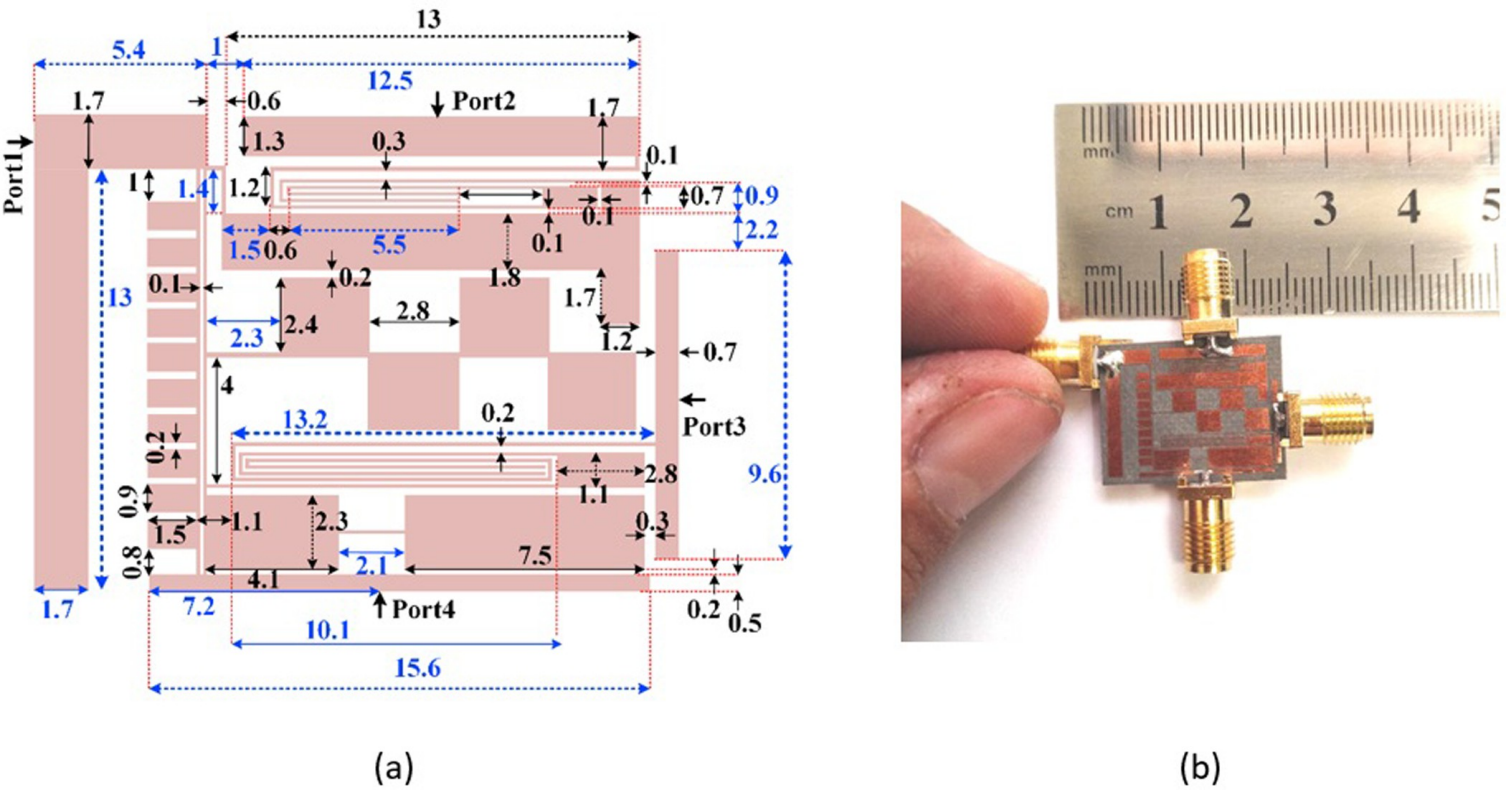
Proposed coupler (a) layout configuration with its dimensions in mm. (b) a photograph of the fabricated coupler.

The layouts and frequency responses of BPF1 and BPF2 are shown in Fig 2, where all dimensions are in mm. A Rogers RT/Duroid5880 substrate with ɛ_r_ = 2.22, tan (δ) = 0.0009 and h = 31 mil is used to design these BPFs. The insertion loss of BPF1 is 0.27 dB at 1.77 GHz, while for BPF2 the insertion loss is 1.2 dB at 1.58 GHz. Since the resonant frequencies are close to each other, these BPFs can be integrated to design a microstrip coupler. During the integration, additional microstrip cells can be added to the coupler structure to improve the frequency response. After obtaining the proposed filters, we must connect them to each other and add the isolation port (Port 4). The function of this port can be controlled by our optimization method. The layout of the proposed coupler with a photograph of the fabricated structure are presented in Fig 3, where all dimensions are in mm. Also, all widths of the thin lines are 0.1 mm. Since our coupler consists of two designed filters. First, we designed two BPFs. Then, we integrated them to achieve our coupler. We didn’t change the dimensions of these filters in the coupler structure. After integrating the proposed BPFs, we added some transition lines (TLs) to the final layout. Reducing the distance between these cells and the main body of the BPFs causes the loading effect. Therefore, we set most of these spaces equal or greater than 0.2 mm. However, their coupling effects are not always negative. Because the simulation results show that, they can improve the return loss somewhat. Since our proposed device is a -3dB coupler, the values of S_21_ and S_31_ at the intersection point is higher than the designed BPFs. However, the best values of S_21_ and S_31_ at the passband are low similar to the BPFs. These filters form the main body of the proposed coupler. Therefore, we focused on designing the presented filters to reach the proposed coupler. This method can be used to design other microstrip filtering devices, which is presented for the first time in this work. The overall size of this coupler is 20.1 mm×14.8 mm = 0.14λ_g_ × 0.103λ_g_, where λ_g_ is the guided wavelength calculated at the operating frequency. The used substrate is a Rogers RT/Duroid 5880 with ɛ_r_ = 2.22, tan (δ) = 0.0009 and h = 31 mil. Four loaded solid rectangles are added on a 0.1 mm thin transmission line between BPF1 and BPF2. This structure plays a role in smoothing the passband as well as suppressing some harmonics. Ports 1 and 4 are connected by ten small solid rectangles loaded on another thin transmission line. This coupler uses a novel curved coupling line as a resonator, which is presented for the first time in this work.

**Fig 4:**
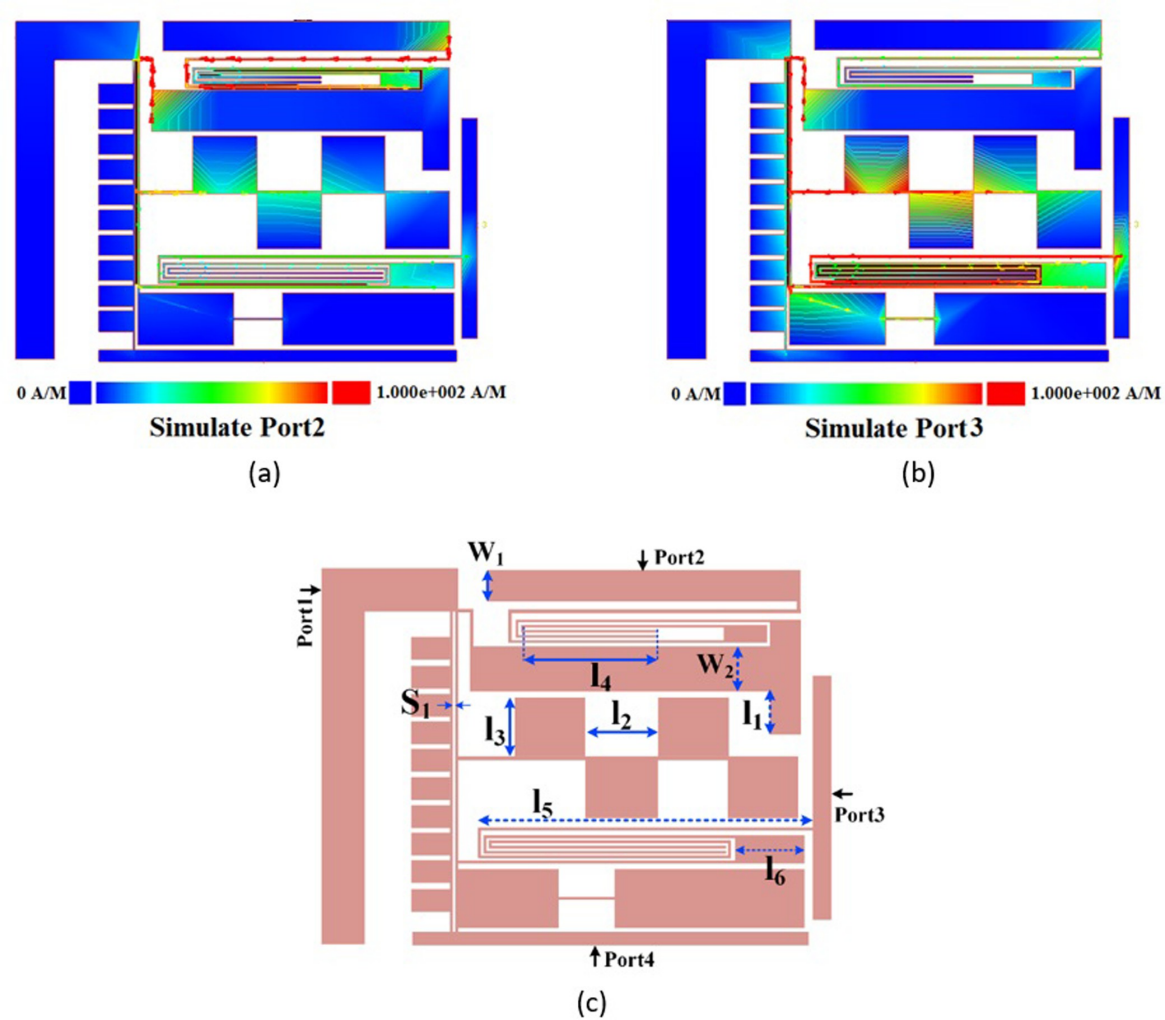
(a) Current density distributions at 1.6 GHz for simulating port 2, (b) current density distributions at 1.6 GHz for simulating port 3, (c) the layout of the proposed coupler with its significant parameters.

**Fig 5:**
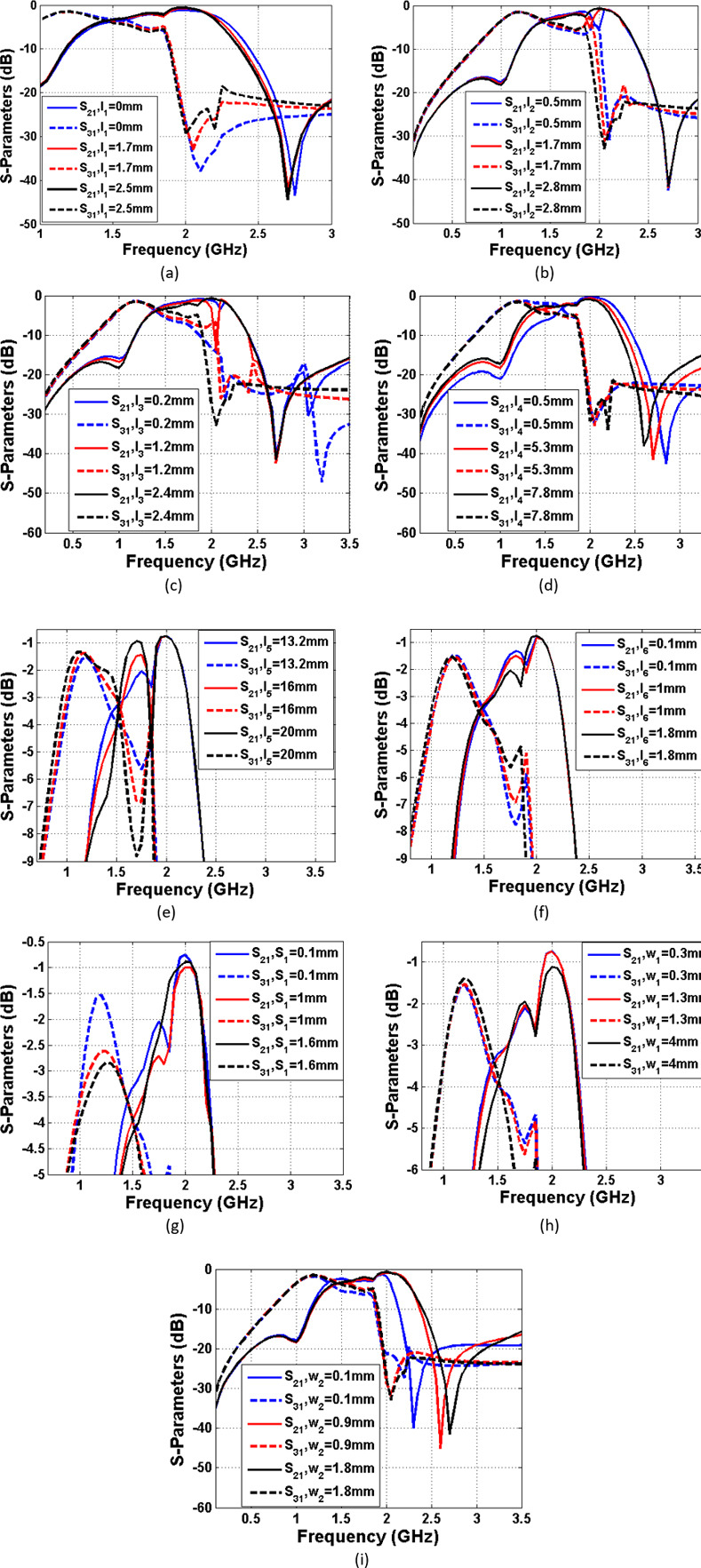
Frequency response of the proposed coupler as functions of the significant parameters, the effect of changing. (a) l_1_ on S_21_ and S_31_, (b) l_2_ on S_21_ and S_31_, (c) l_3_ on S_21_ and S_31_, (d) l_4_ on S_21_ and S_31_, (e) l_5_ on S_21_ and S_31_, (f) l_6_ on S_21_ and S_31_, (g) S_1_ on S_21_ and S_31_, (h) w_1_ on S_21_ and S_31_, (i) w_2_ on S_21_ and S_31_.

**Fig 6:**
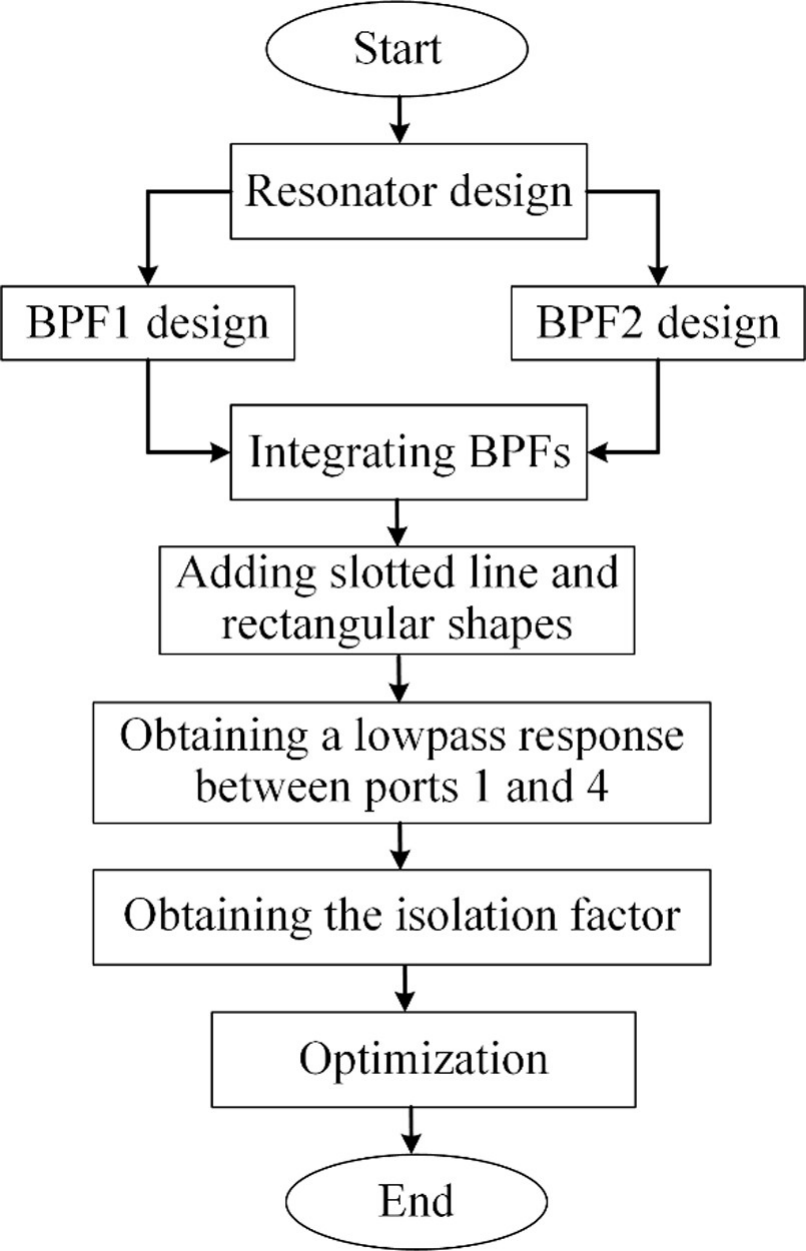
Summarized design steps of the proposed coupler.

To find the important parameters and to facilitate the optimization, the current density distributions (at 1.6 GHz) for the excitation of ports 2 and 3 are shown in Fig 4. Also, the significant physical length and widths are specified in Fig 4. As can be seen, the thin lines have more current densities. Fig 5 depicts the frequency response as functions of nine significant parameters shown in Fig 4. The proposed filters have loading effects after connecting. To reduce the negative effects and improve the frequency response, the rectangular cells are added to the final layout. These cells have capacitive characteristics and their dimensions are selected by the optimization method presented in Fig 5. Some steps of this optimization method is to change the physical dimensions S_1_, l_2_ and l_3_. As shown in Fig 5, increasing the physical lengths l_1_ and l_2_ leads to create some harmonics. By tuning l_3_, l_5_ and l_6_ we can obtain a smoother passband. To shift *S*_21_ to the left and improve the losses, we can increase l_4_ and reduce S_1_ respectively. Another way for shifting *S*_21_ to the left is increasing the width w_2_, while tuning w_1_ has more impact on the loss at port 2. Fig 6 shows the design steps for the proposed coupler. As shown in this figure, after designing the resonator we obtained two BPFs. Next, we integrated them in such a way that the final structure occupies the least possible space. An additional slotted line and some rectangular shapes are added after connecting the presented BPFs. These cells are added to improve the isolation factor. All of the rectangular cells have capacitor feature where they are shunt. When they are placed next to the thin lines with the inductor feature, a lowpass frequency response is created (the stopband of this lowpass filter is located on the passband of our coupler). Accordingly, we can create an isolation port.

### Simulation and measurement results

**Fig 7:**
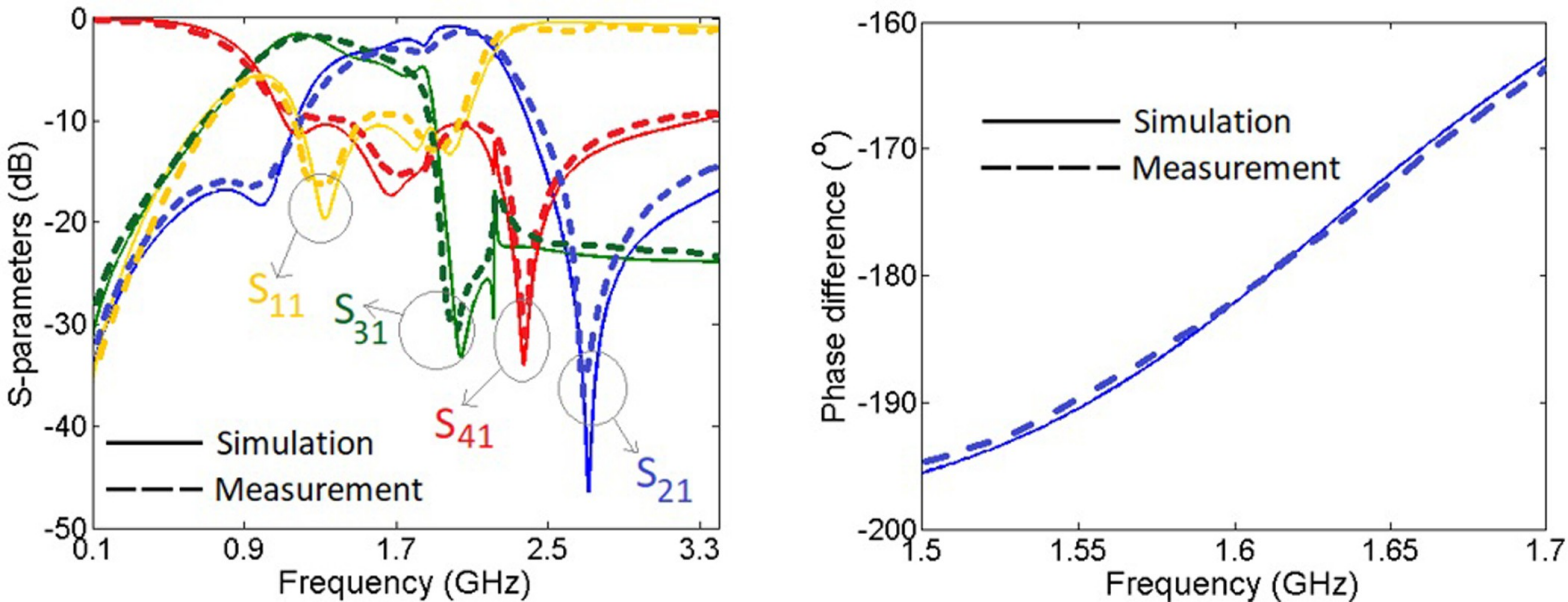
Simulated and measured S-parameters and phase difference.

The simulation results are obtained by the EM simulator of Advanced Design Systems software. The measurement results are obtained by an HP8757A network analyzer. The simulation and measurement results are close to each other. However, due to having copper and terminal losses the measured losses are a little higher than the simulations losses. The simulated and measured S-Parameters and phase difference between *S*_21_ and *S*_31_ are presented in Fig 7.

The operating frequency of this coupler is located at f_o_ = 1.611 GHz, where the phase difference between *S*_21_ and *S*_31_ is only 0.023° at this frequency. The obtained values of *S*_21_ and *S*_31_ are -3.45 dB and -3.75 dB, respectively. There are several transmission poles in *S*_11_ and *S*_41_ (at the passband), which the best values of them are -19.67 dB and -33.99 dB, respectively. In the passband, the best values of S_31_ and S_21_ are -2.275 dB and -2.108 dB, respectively (located at two difference frequencies). However, the magnitude imbalance at the passband is below ±3.5 dB. For the values of *S*_11_ better than -10 dB, the fractional bandwidth (FBW) is 56.1%. Also, from 1.1 GHz to 2.3 GHz both S_21_ and S_31_ are higher than -6 dB. Therefore, the -6dB FBW is near 75%. The phase imbalance from 1.53 GHz to 1.67 GHz is below 10º while near the operating frequency from 1.58 GHz to 1.64 GHz the phase imbalance value is below 5º. The harmonics are suppressed up to 3.5 GHz with a maximum harmonic level of -15.7 dB. The value of S_11_ is better than -10 dB from 1.189 GHz to 2.07 GHz, where the magnitude imbalance at this frequency range is better than ±3.5 dB. This frequency range is suitable for mid-band 5G applications, which covers 1–6 GHz frequency range.

### Comparison with the previous works

To verify the advantages of the proposed coupler, we compared it with the previously reported couplers as shown in Table 1. From this table it is clear that the best values of *S*_21,_ *S*_31_ and FBW% are obtained in this work. Moreover, our coupler size and phase imbalance are very small. Only the proposed coupler in [14] is smaller than our coupler. However, it does not have a filtering frequency response while we could suppress the harmonics up to 2.17*f*_o_. As shown in the comparison table, several of the previous couplers do not have any harmonic attenuations.

**Table 1:** Comparison between our coupler and the previous couplers (PI: Phase Imbalance; FBW: Fractional Bandwidth; FR: Filtering Response; HS: Harmonic Suppression; *: Approximate value).

Refs.	׀*S*_31_׀ (dB)	׀*S*_21_׀ (dB)	PI (degree)	FBW (%)	Size (λ_g_^2^)	FR	HS
Our coupler	3.75	3.45	0.023	56.1	0.014	Yes	2.17f_o_
[10]	3.1	3.1	0.8	---	0.042	No	No
[11]	4.4±0.5	3±1.4	3	3.5	0.138	Yes	1.5f_o_
[12]	3.08	3	0.03	---	0.037	No	No
[13]	3.7	3.3	---	---	---	Yes	2f_o_
[14]	3.1	2.9	0.01	---	0.011	No	No
[15]	8.11	1.38	7.1	---	---	Yes	No
[16]	15.3	0.66	1.36	53.4	0.327	Yes	No
[17]	3.65	2.97	3.6	32.2	0.043	No	No
[18]	3.8	2.9	3	52.3	0.023	Yes	2.1f_o_
[19]	3.28	3.28	0.2	18.8	0.023	Yes	2f_o_
[20]	3.65	3.52	‐‐‐	‐‐‐	0.0231	Yes	No
[21]	3.6±0.5	3.6±0.5	‐‐‐	49	0.2379^*^	Yes	No
[22]	-3±0.8	-3±1	3	‐‐‐	0.307*	Yes	No
[23]	Better than 4	Better than 4	5	‐‐‐	‐‐‐‐	No	No
[24]	3.6	3.58	2.8	‐‐‐	0.302*	No	No
[25]	3±1.6	3±2	10	34.4	‐‐‐	No	No

## Conclusion

A microstrip coupler with a novel structure, good performance and compact size is presented in this work. We used a meandrous coupled line resonator to achieve a new coupler configuration. We analyzed the proposed resonator to study its behavior. This coupler operates at 1.611 GHz and its size is only 297.5 mm^2^. The proposed coupler is fabricated on a substrate with 2.22 dielectric constant and 31 mil thickness and then it is measured. To verify the advantages of this coupler, a complete comparison with the previously reported works is done. Having filtering frequency response, low losses in the passband, wide fractional bandwidth (FBW) and balanced phases are the other advantages of this work. Also, our coupler has reasonable return loss and isolation. None of the previous couplers has these advantages at the same time. Therefore, it can be easily used in designing high-performance RF communication systems.
